# Connexin32 gap junction channels deliver miR155-3p to mediate pyroptosis in renal ischemia-reperfusion injury

**DOI:** 10.1186/s12964-023-01443-3

**Published:** 2024-02-12

**Authors:** Liubing Chen, Hongyi Fang, Xiaoyun Li, Peiling Yu, Yu Guan, Cuicui Xiao, Zhizhao Deng, Ziqing Hei, Chaojin Chen, Chenfang Luo

**Affiliations:** Department of Anesthesiology, The Third Affiliated Hospital of Sun Yat-sen University, No. 600 Tianhe Road, Guangzhou, 510630 Guangdong Province China

**Keywords:** Renal ischemia reperfusion injury, Hypoxia and reoxygenation injury, Connexin32, Pyroptosis, miR155-3p

## Abstract

**Objectives:**

To explore whether the gap junction (GJ) composed by connexin32(Cx32) mediated pyroptosis in renal ischemia-reperfusion(I/R) injury via transmitting miR155-3p, with aim to provide new strategies for the prevention and treatment of acute kidney injury (AKI) after renal I/R.

**Methods:**

8–10 weeks of male C57BL/ 6 wild-type mice and Cx32 knockdown mice were divided into two groups respectively: control group and renal I/R group. MCC950 (50 mg/kg. ip.) was used to inhibit NLRP3 in vivo. Human kidney tubular epithelial cells (HK - 2) and rat kidney tubular epithelial cells (NRK-52E) were divided into high-density group and low-density group, and treated with hypoxia reoxygenation (H/R) to mimic I/R. The siRNA and plasmid of Cx32, mimic and inhibitor of miR155-3p were transfected into HK - 2 cells respectively. Kidney pathological and functional injuries were measured. Western Blot and immunofluorescent staining were used to observe the expression of NLRP3, GSDMD, GSDMD-N, IL - 18, and mature IL-18. The secretion of IL-18 and IL-1β in serum, kidney tissue and cells supernatant were detected by enzyme-linked immuno sorbent assay (ELISA) kit, and the expression of NLPR3 and miR155-3p were detected by RT-qPCR and fluorescence in situ hybridization (FISH).

**Results:**

Tubular pyroptosis were found to promote AKI after I/R in vivo and Cx32-GJ regulated pyroptosis by affecting the expression of miR155-3p after renal I/R injury. In vitro, H/R could lead to pyroptosis in HK-2 and NRK-52E cells. When the GJ channels were not formed, and Cx32 was inhibited or knockdown, the expression of miR155-3p was significantly reduced and the pyroptosis was obviously inhibited, leading to the reduction of injury and the increase of survival rate. Moreover, regulating the level of miR155-3p could affect survival rate and pyroptosis in vitro after H/R.

**Conclusions:**

The GJ channels composed of Cx32 regulated tubular pyroptosis in renal I/R injury by transmitting miR155-3p. Inhibition of Cx32 could reduce the level of miR155-3p further to inhibit pyroptosis, leading to alleviation of renal I/R injury which provided a new strategy for preventing the occurrence of AKI.

Video Abstract

**Supplementary Information:**

The online version contains supplementary material available at 10.1186/s12964-023-01443-3.

## Introduction

Acute kidney injury (AKI) manifests as the rapid decline of renal function, and accumulation of body metabolic toxins along with multiple organs failure. It has remained a high morbidity of AKI around the world over the past decades, with more than 2 million people dying from AKI worldwide, which brings a huge burden to society medical resource [1–3]. As one of the most significant reasons leading to AKI, renal ischemia reperfusion (I/R) is induced by several pathological processes such as shock, circulatory failure, kidney transplantation and renal operations, and furtherly, tubular cells regulated necrosis and amplified inflammation response are proved to be major mechanisms for incidence and development of I/R and AKI [4, 5].

Regulatory necrosis is considered as the main form of cell death in kidney injury and kidney disease, including necroptosis, pyroptosis, and ferroptosis [6–10]. Pyroptosis, a type of pro-inflammatory programmed cell death, is also known as Gasdermin D (GSDMD) -mediated programmed necrosis, caused by disturbances in extracellular or intracellular homeostasis associated with innate immunity [11]. In detail, in response to harmful stimuli, toll-like receptors (TLRs) of multiple cells activate assembly of multiple-protein complexes and NLR family pyrin domain containing 3 (NLRP3)- inflammasome, which induce the activation and maturation of caspase-1, leading to the proteolytic maturation of the pro-inflammatory cytokines Interleukin(IL)-1β and IL-18, as well as the pore-forming protein GSDMD, and the resulting N-terminal fragment (GSDMD-N) then undergoes a conformational change, oligomerizes, and binds to the cell membrane which forms pyroptotic pores on the surface of cell membrane and therefore actives release of mature proinflammatory cytokines such as IL-1β and IL-18 [12, 13]. It should be noticed that studies have suggested tubular pyroptosis contributed to severe AKI [14–16]. The earliest study in 2014 found that renal I/R injury induce the pyroptosis of renal tubule epithelial cells via the CHOP-caspase-11 pathway [17]. Besides, more severe AKI occurred after renal I/R in diabetic rats, accompanied by increased NLRP3/caspase1/caspase11/IL-1β/IL-18 signals [18]. Another study also showed that pretreatment with β-hydroxybutyrate could be a novel strategy against AKI for its anti-pyroptosis effect [19]. It seems that pyroptosis leads to amplified renal injury after I/R through both direct cell death and aggravation of inflammation by releasing pro-inflammatory cytokines. However, regulation of tubule pyroptosis during AKI was complex and undefined.

Gap junction (GJ) is a type of cellular channel for information exchange mediated the delivery of ions and small molecules less than 1 kDa between tubular cells, such as calcium, triphosphate, reactive oxygen species (ROS), glutathione and microRNA. This is called the paracellular effect or bystander-effect that plays an important role in cell homeostasis, tissue growth, and differentiation in multiple organs [20, 21]. More than 90% of GJs in kidney tubules are composed of Connexin-32(Cx32) proteins. Our previous studies found that bystander-effect by Cx32-GJ among tubule epithelial cells (TECs) aggravated apoptosis signaling and oxidative stress response during reanl I/R induced-AKI [22, 23], which was modified by transmission and distribution of reactive oxygen species (ROS). GJ channels had been reported to be involved in regulation of different types of cell death in various physiological process, including cell pyroptosis [24]. However, the role of Cx32-GJ on cell pyroptosis during AKI had not been reported so far and which kind of molecule was delivered to mediate the process needs specific exploration.

“microRNA”(miRNA) is a RNA sequence of about 22 nucleotides that promote or restrain gene expression and mRNA translation on a post-transcriptional level [25, 26]. Certain errors on regulation of miRNA lead to kinds of human diseases including cancer, liver complaint, and kidney disorders [27–29]. It had been found that downregulation of miR155 level prevented kidney tissue from inflammation and injury in sepsis-induced AKI [30]. Furthermore, miR155 was proved to facilitate TECs pyroptosis both in vitro and in vivo [31]. Each miRNA is defined as a miRNA duplex comprising two strands (5p and 3p) and after strand selection only one strand becomes active and is selectively incorporated into the RNA-induced silencing complex to be functional miRNA finally [32, 33]. Interestingly, it was recently found that human adipose tissue stem cells (hASCs)-derived exosomal miR-155-5p inhibited pyroptosis of nucleus pulposus cells and alleviated intervertebral disc degeneration [34]. What’s more, miR155-3p was reported to elevate activation of NLRP3 inflammasome and inflammatory response in murine microglial cells [35]. Recent evidences also showed that miR155-3p induced cytokines expression in human dendritic cells, macrophages, and T cells through rapid immune response to injury stimulation at the early stage of inflammation [36–38]. However, the precise role of miR155-3p in tubular pyroptosis and AKI was still undefined. In the current study, we hypothesized that renal pyroptosis took part in amplification of AKI after renal I/R, which was regulated by signals of Cx32-GJ via modulating the transfer of miR155-3p among tubular cells. By establishing model of renal I/R injury of mice and H/R injury of TECs, it was shown that TECs pyroptosis was initiated after AKI caused by renal IR, and depression of TECs pyroptosis contributed to attenuated AKI and enhanced HK-2 and NRK-52E cells survival. What’s more, inhibition of Cx32-GJ and knockdown of Cx32 reduced tubular pyroptosis, AKI, and the level of miR155-3p in kidney tissue at the same time. Downregulation of miR155-3p prevented kidney tubular cells from pyroptosis and inflammation, which suggested that Cx32-GJ regulated tubular pyroptosis via transmitting of miR155-3p. Our results confirmed the bystander-effect of Cx32-GJ in AKI and TECs pyroptosis via regulation of miR155-3p signals which was a potential target of AKI treatment.

## Result

### Pyroptosis was aggravated in mice during I/R-induced AKI

**Fig. 1:**
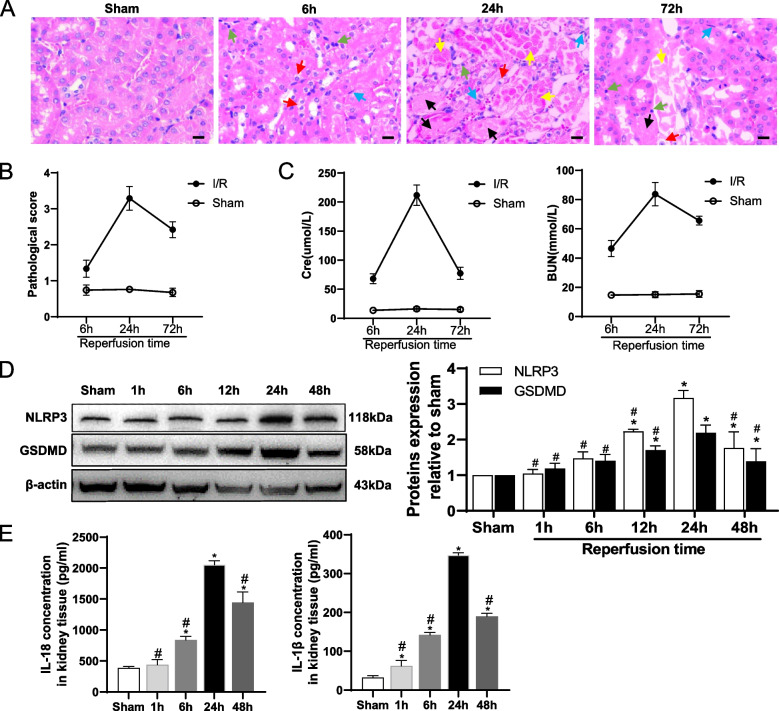
Pyroptosis was aggravated in mice during I/R-induced AKI. **A** Representative images of hematoxylin–eosin staining (H&E)-stained kidney sections. Red arrow: cell swelling and cytoplasm rarefaction; Yellow arrow: tubular necrosis; Black arrow: cast formation; Green arrow: karyopyknosis; Blue arrow: inflammatory cells. Scale bar = 50 μM. **B** Kidney injury was scored based on the percentage of tubules displaying tubular dilation, tubular necrosis, and cast formation using a scale from 0 (normal) to 4 (≥75%) (*n* = 5). **C** Serum Cr and BUN levels were measured using enzyme-linked immunosorbent assay (ELISA) (*n* = 5). **D** NLRP3 and GSDMD expression were analyzed by Western blots using kidney lysates from sham-operated mice and mice at different renal reperfusion time points. * *p* < 0.05 vs. sham; # *p* < 0.05 vs. mice subjected to renal reperfusion for 24 hours. Data are presented as means ± SEM (*n* = 3). **E** Concentration of IL-1β and IL-18 in kidney tissue was analyzed by ELISA at different reperfusion time points. * *p* < 0.05 vs. sham. # *p* < 0.05 vs. mice subjected to renal reperfusion for 24 hours. Data are presented as means ± SEM (*n* = 5)

We first used a model of renal I/R to examine the dynamic process of AKI. As similarly to our previous findings, histologic analysis demonstrated kidney injury peaked at 24 h after reperfusion, and then declined (Fig. 1A, B). Meanwhile, the changes of both serum creatinine (Cr) and blood urea nitrogen (BUN) mirrored the patterns of pathological injury, reaching peak at 24 h of reperfusion and then began to recover (Fig. 1C).

NLRP3, the inflammasome sensor, as well as GSDMD,and the released inflammatory cytokines IL-1β and IL-18 have been identified as the specific molecular markers for pyroptosis. We examined the expression of NLRP3 and GSDMD in the I/R-induced AKI mice. The expression of NLRP3 and GSDMD were significantly increased in I/R-treated mice. To note, they peaked at 24 h after reperfusion and then began to decline (Fig. 1D). Similarly, the levels of IL-18 and IL-1β were also significantly increased in mice after I/R treatment (Fig. 1E). These findings suggested pyroptosis in mice after I/R treatment were also a dynamic process, which is in consistent with the trend of kidney pathological injury.

### Inhibition of pyroptosis alleviated I/R and H/R injury in vivo and in vitro

**Fig. 2:**
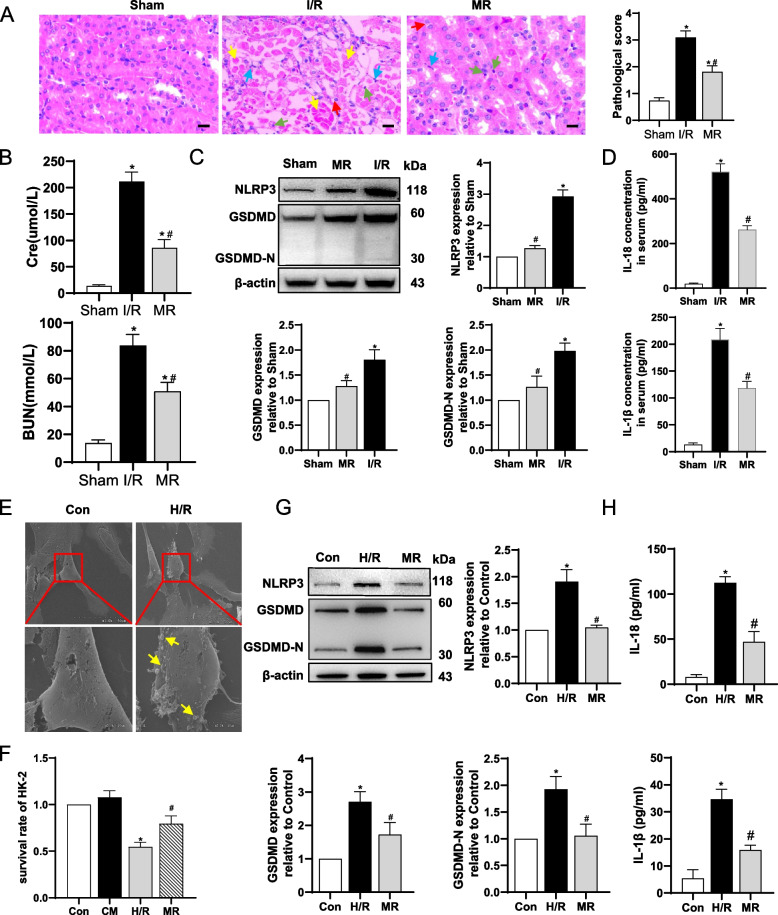
Inhibition of pyroptosis alleviated I/R and H/R injury in vivo and vitro. Kidneys and blood samples were collected from mice 24 hours after renal reperfusion following renal I/R. HK-2 cells were seeded at high density (125,000 cells/cm^2^, GJ formed). Cells in the hypoxia/reoxygenation (H/R) group were subjected to 24 hours of hypoxia followed by 4 hours of reoxygenation. **A** Representative H&E-stained kidney sections and kidney injury scores. Red arrow: cell swelling and cytoplasm rarefaction; Yellow arrow: tubular necrosis; Black arrow: cast formation; Green arrow: karyopyknosis; Blue arrow: inflammatory cells. Scale bar = 50 μM. * *p* < 0.05 vs. sham; # *p* < 0.05 vs. I/R. Data are means ± SEM (*n* = 5). **B** Serum samples were collected for measurements of serum creatinine and BUN levels. * *p* < 0.05 vs. sham; # *p* < 0.05 vs. I/R. Data are means ± SEM (*n* = 5). **C** Western blot analysis of NLRP3, GSDMD, and GSDMD-N expression using kidney lysates. * *p* < 0.05 vs. sham; # *p* < 0.05 vs. I/R mice. Data are means ± SEM (*n* = 3). **D** Concentration of IL-18 and IL-1β in serum analyzed by ELISA. * *p* < 0.05 vs. sham; # *p* < 0.05 vs. I/R. Data are means ± SEM (*n* = 5). **E** Morphology of HK-2 cells in different groups under scanning electron microscopy. Yellow arrow: bursting extensions. Scale bar = 10 μM. **F** Survival rate of HK-2 cells in different groups at high density. * *p* < 0.05 vs. control; # *p* < 0.05 vs. H/R. Data are means ± SEM (*n* = 5). **G** Western blot analysis of NLRP3, GSDMD, and GSDMD-N expression in HK-2 cells. * *p* < 0.05 vs. control; # *p* < 0.05 vs. H/R. Data are means ± SEM (*n* = 3). **H** Concentration of IL-18 and IL-1β in culture supernatant of HK-2 cells in different groups. * *p* < 0.05 vs. control; # *p* < 0.05 vs. H/R. Data are means ± SEM (*n* = 5)

To investigate the effects of NLRP3 on I/R-induced AKI, we pretreated mice with the NLRP3 inhibitor MCC950. Histologic analysis demonstrated that I/R-induced tubular dilation, loss of brush border, cast formation, and cell lysis in tubules were significantly improved in mice pretreated with MCC950 (Fig. 2A), accompanied by a decrease of serum BUN and Cr levels (Fig. 2B). In addition, western blotting revealed MCC950 application could alleviate renal pyroptosis by decreasing the expression of NLRP3, GSDMD, GSDMD-N, IL-18 and mature IL-18 in I/R mice (Fig. 2C, Supplementary Fig. S1A). Consistently, serum levels of inflammatory cytokines IL-18 and IL-1β were significantly reduced (Fig. 2D).

In vitro, human kidney tubular epithelial cells (HK-2) were exposed to hypoxia for 24 h and reoxygenation for 4 h (H/R). As Fig. 2D showed, HK-2 cells exhibited a round shape with bursting extensions, the typical morphological change of pyroptosis in cells after H/R treatment. The survival rate of cells increased significantly when HK-2 cells were pretreated with MCC950 for 1 h before hypoxia (Fig. 2F). What’s more, the expression of NLRP3, GSDMD, GSDMD-N, IL-18 and mature IL-18 were significantly increased after H/R injury, whereas MCC950 decreased the proteins levels (Fig. 2G, Supplementary Fig. S1B). Similarly, the levels of IL-18 and IL-1β were increased in HK-2 cells supernatant after H/R, and MCC950 could reverse that (Fig. 2H).

These results suggested that inhibition of NLRP3 attenuated renal injury in mice after I/R and protected HK-2 cells from H/R.

### Knockout of Cx32 suppressed NLRP3-mediated pyroptosis in mice with I/R-induced AKI

**Fig. 3:**
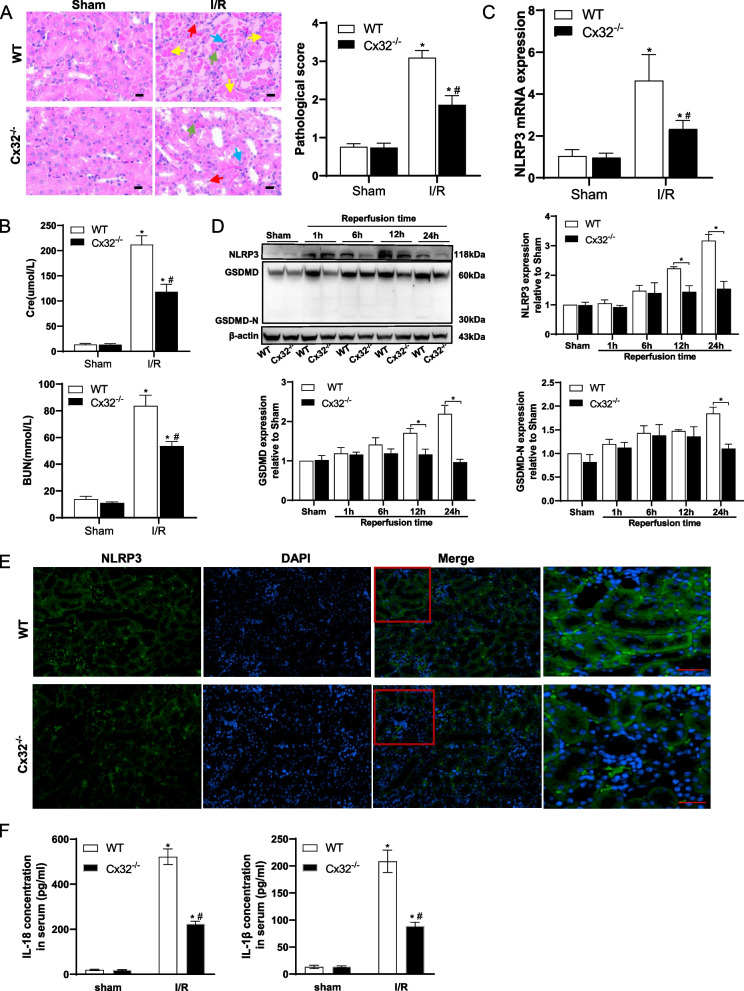
Cx32 knockout suppressed NLRP3-mediated pyroptosis in mice with I/R-induced AKI. **A** Representative H&E-stained kidney sections and kidney injury scores. Scale bar = 50 μM. WT: wild-type; I/R: renal ischemia/reperfusion; Cx32^−/−^: Cx32 knockout mice. * *p* < 0.05 vs. sham; # *p* < 0.05 vs. WT + I/R group. Data are means ± SEM (*n* = 5). **B** Serum creatinine and BUN levels in different groups. * *p* < 0.05 vs. sham; # *p* < 0.05 vs. WT + I/R group. Data are means ± SEM (*n* = 5). **C** RT-qPCR analysis of NLRP3 expression in renal tissue. * *p* < 0.05 vs. sham; # *p* < 0.05 vs. WT + I/R group. Data are means ± SEM (*n* = 5). **D** Western blot analysis of NLRP3, GSDMD, and GSDMD-N expression in kidney lysates at different reperfusion times. * *p* < 0.05 vs. WT + I/R group. Data are means ± SEM (*n* = 3). **E** Immunofluorescence results of NLRP3 expression in kidneys reperfused for 24 h. Scale bar = 50 μM. **F** Concentration of IL-18 and IL-1β in serum analyzed by ELISA. * *p* < 0.05 vs. sham; # *p* < 0.05 vs. WT + I/R group. Data are means ± SEM (*n* = 5)

Our previous studies showed that Cx32 GJ channels enhanced intercellular ROS transmission in AKI induced by renal I/R [22]. To explore whether Cx32 GJ was involved in NLRP3 mediated pyroptosis during AKI, we utilized Cx32 knockout (Cx32^−/−^) mice to establish an I/R model. Consistent with our previous findings, both the pathological scores and the levels of Cr and BUN in serum were significantly reduced in Cx32^−/−^ mice (Fig. 3A, B). Importantly, the knockdown of Cx32 gene resulted in a decrease in NLRP3 mRNA levels in the kidney after I/R (Fig. 3C). Western blot analysis revealed a decrease in the expression of NLRP3, GSDMD, and GSDMD-N in Cx32^−/−^ mice, particularly at 24 h after reperfusion, compared to wild-type (WT) mice (Fig. 3D). Consistently, immunofluorescence results also demonstrated a significant suppression of NLRP3 expression in Cx32^−/−^ mice following I/R (Fig. 3E). Furthermore, the levels of IL*-*18 and IL*-*1β were also significantly reduced by Cx32 knockout (Fig. 3F). These results suggested Cx32 affected tubular pyroptosis and knockout of Cx32 suppressed NLRP3-mediated pyroptosis in I/R-induced AKI.

### Inhibition of Cx32 channel function attenuated NLRP3-mediated pyroptosis in H/R injury

**Fig. 4:**
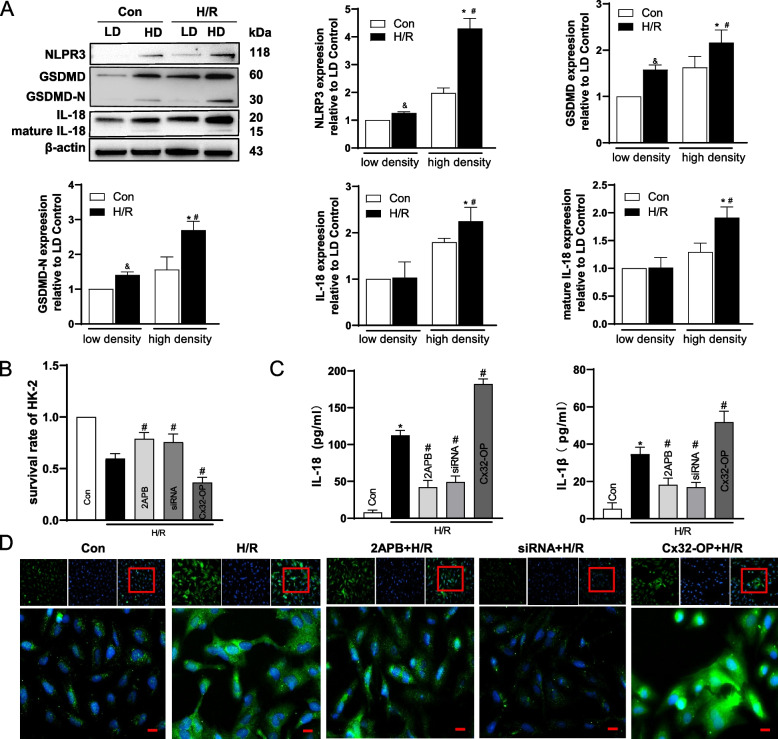
Inhibition of Cx32 channel function attenuated NLRP3-mediated pyroptosis in H/R injury. HK-2 cells were seeded at low density (25,000 cells/cm^2^, no GJ formed) and high density (125,000 cells/cm^2^, GJ formed) and subjected to hypoxia/reoxygenation (H/R) treatment. **A** Western blot analysis of NLRP3, GSDMD, GSDMD-N, IL-18, and mature IL-18 in HK-2 cells. LD: low density group; HD: high density group. *p < 0.05 vs. control group at high density; # *p* < 0.05 vs. H/R group at low density; & *p* < 0.05 vs. control group at low density. Data are means ± SEM (*n* = 3). **B** Survival rate of HK-2 cells at high density in different groups. * *p* < 0.05 vs. control; # *p* < 0.05 vs. H/R. Data are means ± SEM (*n* = 5). **C** Concentration of IL-18 and IL-1β in culture supernatant of HK-2 cells at high density in different groups. * *p* < 0.05 vs. control; # *p* < 0.05 vs. H/R. Data are means ± SEM (*n* = 5). **D** Immunofluorescence results of NLRP3 expression in HK-2 cells at high density. Scale bar = 50 μM. 2APB: cells were pretreated with 2APB (25 μM) for 1 h before the next step; siRNA: Cx32-siRNA (50 nM) transfected into cells for 48 h before the next step. Cx32-OP: the cells were transfected with plasmid-Cx32 for 48 hours to achieve overexpression of Cx32 before the next step

In vitro, three different methods were employed to alter GJ function composed of Cx32 in HK-2 cells. First, HK-2 cells were seeded at low-density (25,000 cells/cm^2^, no GJ formed) and high-density cell culture (125,000 cells/cm^2^, GJ formed), respectively. Result showed that when exposed to H/R, HK-2 cells growth declined at both low-density and high-density cell culture. Interestingly, cell damage was more pronounced at high-density culture (Supplementary Fig. S2). When GJ was formed at high-density cell culture, cells pyroptosis aggravated with higher expression of NLRP3, GSDMD, GSDMD-N, IL-18 and mature IL-18 after H/R, compared with cells seeded at low-density cell culture (Fig. 4A). Second, 2-Aminoethoxydiphenyl borate (2APB), an inhibitor of Cx32, and Cx32-siRNA were employed to inhibit GJ channels composed by Cx32. Both 2-APB and Cx32-siRNA had no significant effect on the survival rate in the control group (Supplementary Fig. S3A). However, 2APB or Cx32-siRNA pretreatment increased HK-2 cells survival rate at high-density cells culture when exposed to H/R (Fig. 4B). Additionally, the levels of IL-18 and IL-1β in supernatant (Fig. 4C), as well as the expression of NLRP3, GSDMD, GSDMD-N, IL-18 and mature IL-18 (Supplementary Fig. S4) were markedly reduced by 2APB or Cx32-siRNA treatment. Immunofluorescence results further demonstrated that NLRP3 expression decreased when pretreat with 2APB or Cx32-siRNA (Fig. 4D). To further confirm the role of Cx32-GJ in NLRP3-mediated pyroptosis, plasmid-Cx32 was used to generate the overexpression of Cx32 (Cx32-OP) in HK-2 cells. As showed in Fig. 4B-D, overexpression of Cx32 reduced the survival rate, while increasing NLRP3 expression, IL-18 and IL*-*1β levels in HK-2 cells after H/R treatment.

Rat kidney tubular epithelial cells (NRK-52Es) were also used in the assay. Similarly, when NRK-52Es were cultured at high density, cellular expression of NLRP3, GSDMD, GSDMD-N, IL-18 and mature IL-18 after H/R were significantly greater than seeded at low-density cell culture with depressed cell survival rate after H/R (supplementary Fig. S5A, B). Moreover, elevations of these proteins after H/R were significantly suppressed when pretreated with 2APB while cell survival was upregulated (Supplementary Fig. S5C, D). The results of the two kinds of TECs in vitro experiments showed that inhibition Cx32 channel function attenuated NLRP3-mediated pyroptosis in H/R injury.

### Cx32 channels regulated the content of miR155-3p induced by I/R or H/R injury

**Fig. 5:**
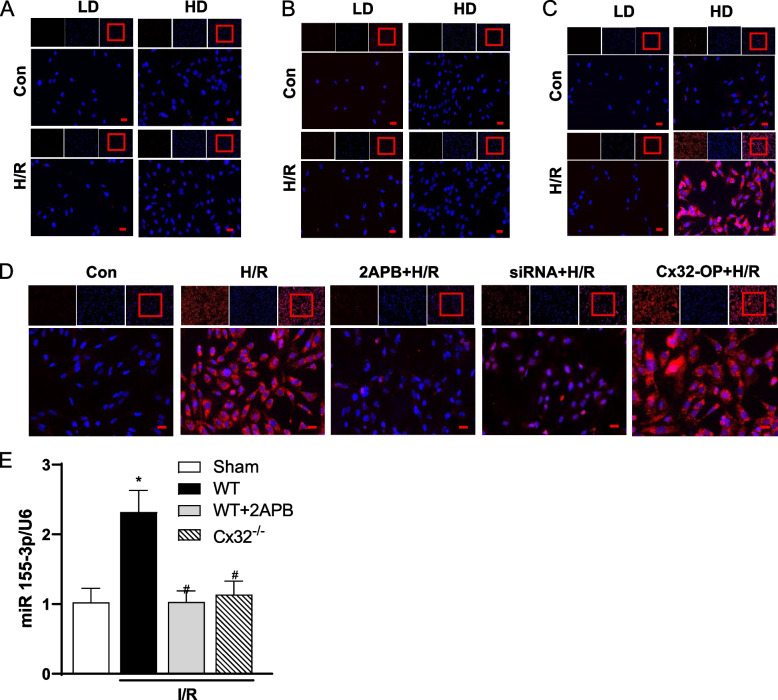
Cx32 channels regulated the level of miR155-3p induced by I/R or H/R injury. HK-2 cells were seeded at low (25,000 cells/cm^2^) or high (125,000 cells/cm^2^) density and subjected to H/R. Bar = 50 μM. **A**-**C** Fluorescence in situ hybridization (FISH) was used to observe the level of miR155 (**A**), miR155-5p (**B**), and miR155-3p (**C**) in HK-2 cells in different groups. Con: control group; LD: low density group; HD: high density group. **D** FISH was used to observe the level of miR155-3p in HK-2 cells at high density in different groups. Con: control group; 2APB: cells were pretreated with 2APB (25 μM) for 1 h before the next step; siRNA: Cx32-siRNA (50 nM) was transfected into cells for 48 h before the next step. Cx32-OP: cells were transfected with plasmid-Cx32 for 48 hours to achieve overexpression of Cx32 before the next step. **E** Quantitative real-time PCR (RT-qPCR) was used to analyze the expression level of miR-155-3p in renal tissue. WT: wild-type; I/R: ischemia reperfusion; 2APB: mice pretreated with 2APB (inhibitor of Cx32, 20 mg/kg, i.p.) for 1 h before renal ischemia; Cx32^−/−^: Cx32 knockout mice. * *p* < 0.05 vs. sham; # *p* < 0.05 vs. the WT + IR group. Data are means ± SEM (*n* = 5)

The results above confirmed that inhibition of Cx32 channel function could alleviate I/R or H/R induced renal injury by regulating NLRP3-mediated pyroptosis, which was always related to the alteration of signal transduction through Cx32 channels. MiR155, an inflammation-related gene, is one of the signals that can be transmitted through Cx32 channels. MiR155-3p or miR155-5p, which are the mature fragments of miR-155, are thought to primarily contribute to inflammation and kidney injury. Therefore, in this section, we investigated the effects of Cx32 channels on the content of miR155, miR155-3p and miR155-5p. Firstly, we aimed to verify which fragment of miR-155 acted as a regulator in the H/R injury. HK-2 cells were seeded at low and high density and the levels of miR155-3p, miR155-5p and miR-155 were detected by FISH. As shown in Fig. 5A and B, the level of both miR155 and miR155-5p didn’t change significantly at the two different densities, regardless of whether the cells suffered from H/R or not. Interestingly, the level of miR155-3p in high density H/R group increased significantly, compared with the control group at high density. However, the level of miR155-3p did not change significantly at low density compared with the control group (Fig. 5C). These results suggested Cx32 channels regulate miR155-3p, not miR155-5p or miR-155, to mediate bystander effect and aggravate H/R injury. Moreover, when HK-2 cells were seeded at high density, both 2APB and Cx32-siRNA pretreatment decreased the expression of miR155-3p obviously after H/R treatment comparing with the control group, indicating Cx32 GJ channels could regulate the transmission of miR155-3p between adjacent cells insulted by H/R (Fig. 5D).

Besides, The RT-qPCR results showed that the level of miR155-3p increased significantly in WT mice after I/R compared with sham group. However, when pretreated with 2APB, or in the Cx32^−/−^ mice, the level of miR155-3p decreased significantly compared with WT mice after IRI (Fig. 5E).

### miR155-3p promoted NLRP3-mediated pyroptosis in H/R injury

**Fig. 6:**
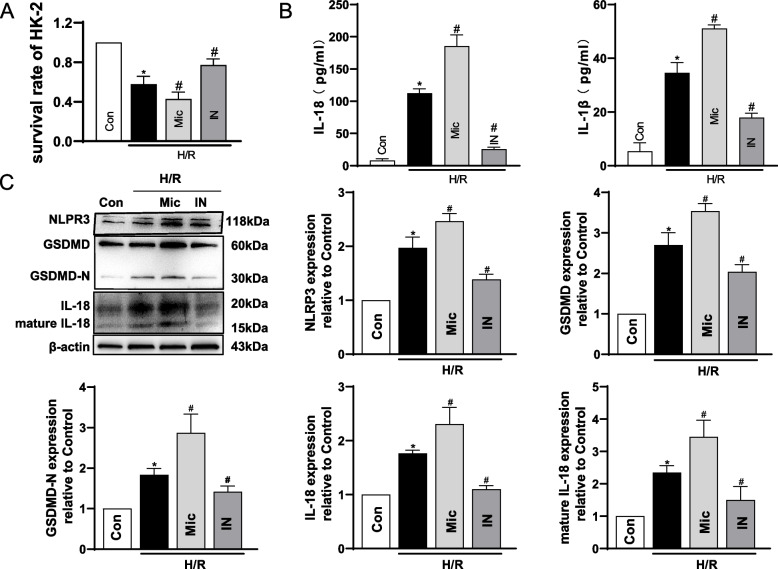
miR155-3p promoted NLRP3-mediated pyroptosis in H/R injury. HK-2 cells were seeded at high density (125,000 cells/cm^2^, GJ formed) and subjected to H/R. **A** The survival rate of HK-2 cells in different groups. Con: control group; Mic: mimic of miR155-3p (50 nM) transfected into cells for 48 h before the next step; IN: inhibitor of miR155-3p (100 nM) transfected into cells for 48 h before the next step. * *p* < 0.05 vs. control; # *p* < 0.05 vs. H/R. Data are means ± SEM (*n* = 5). **B** Concentration of IL-18 and IL-1β in culture supernatant in HK-2 cells of different groups. * *p* < 0.05 vs. control; # *p* < 0.05 vs. H/R. Data are means ± SEM (*n* = 5). **C** The expression of NLRP3, GSDMD, GSDMD-N, IL-18, and mature IL-18 in HK-2 cells were analyzed by western blots. * *p* < 0.05 vs. control; # *p* < 0.05 vs. H/R. Data are means ± SEM (*n* = 3)

To investigate the role of miR155-3p in NLRP3-mediated pyroptosis during the H/R injury of renal TECs, the mimic and inhibitor of miR155-3p were transfected into HK-2 cells for further study. As anticipated, the mimic of miR155-3p decreased the survival rate of HK-2 cells exposed to H/R, while the inhibitor increased it (Fig. 6A). Levels of IL-1β and IL-18 levels were elevated in HK-2 cells supernatant pretreated with the mimic and significantly decreased by the inhibitor of miR155-3p (Fig. 6B). Similar changes were observed in GSDMD, GSDMD-N, IL-18 and mature IL-18 expression in HK-2 cells, along with the expression of NLRP3 when pretreated with the mimic or the inhibitor of miR155-3p respectively (Fig. 6C). Immunofluorescence results also demonstrated that elevation in NLRP3 expression of HK-2 cells after H/R injury was significantly promoted by the mimic and suppressed by the inhibitor of miR155-3p (Supplementary Fig. S6). These results demonstrated that miR155-3p promoted NLRP3-mediated pyroptosis in HK-2 cells exposed to H/R injury.

## Materials and methods

### Materials

2APB was purchased from Sigma-Aldrich. MCC950 was provided by MCE. IL-18 and IL-1β ELISA Kit were purchased from Abclonal. MicrOFF™ has-miR-155-3p mimic, micrOFF™ has-miR-155-3p inhibitor, plamid-Cx32 and Cx32-siRNA were obtained from Ribobio. FBS was offered by Gibco. The Keratinocyte-SFM medium was purchased from ThermoFisher. All-in-One miRNA RT-qPCR Detection Kit was provided by Ribobio. Lipo8000™ transfection reagent was purchased from Beyotime.

### Animals

Male WT (C57BL/6) mice (aged 8–10 weeks, 20-25 g) were purchased from Changsheng Biotechnology Co., LTD. Cx32^−/−^ male mice (aged 8–10 weeks, 20-25 g, on the background of C57BL/6) were provided by Emma Chemicals, Inc. (B6.129S4-Gjb1tm1Kwi/Cnrm, No. 00243). All the protocols and design involved animals were with the approval of the Institutional Animal Care and Use Committee at The Third Affiliated Hospital of Sun Yat-Sen University (Guangzhou, China). Animal care was in accordance with guidelines of Guide for the Care and Use of Laboratory Animals (National Insti-tutes of Health 1985). Animals were raised in the standard conditions (25–27 °C with 12 h light/dark cycle), and allowed to get access to pathogen-free laboratory food and distilled water freely.

### Renal I/R models

We used an established model of renal I/R injury. Briefly, mice were anesthetized with intraperitoneal injections of ketamine (60 mg/kg), then the bilateral renal pedicles were clamped by atraumatic microvascular clamp (Fine Science Tools, North Vancouver, British Columbia, Canada) to construct the model of ischemia. The clamps were loosened after 45 min to restore the blood flow of renal. Mice in the sham group underwent the identical surgery manner without clamping the renal pedicles. During the surgery, mice were place on the heating pad to maintain a constant body temperature at 37 °C. After surgery, mice were placed in the microisolator cages, allowing get access to chow and water freely. Mice were sacrificed at different time points (1 h,6 h,12 h,24 h,48 h, and 72 h) after reperfusion, while the blood and kidneys were collected for further experiments. According to the earlier report [39], WT mice in MR group were pretreated with intraperitoneal injection of 50 mg/kg MCC950 for 1 h, and then underwent renal ischemia surgery and sacrificed at 24 h after I/R. Group of 2APB were wild type mice pretreated with 2APB (inhibitor of Cx32, 20 mg/kg, i.p.) for 1 h before renal ischemia and sacrificed at 24 h after I/R.

### Histology

Kidneys were fixed in 10% buffered formalin before embedding in paraffin. The kidney tissue was cut into 5 μm thick sections for H&E to analyze the histopathological pattern change. According to the report [23], the injuries of kidney tubules, including tubular epithelial swelling, necrosis, and cast formation, were graded with a score of 0–4 (0, means no change; 1, means change affecting < 25% of the field; 2, change affecting between 25 and 50% of the field; 3, change affecting between 50 and 75% of the field; 4, change affecting > 75% of the field). Ten photographs were taken for each kidney tissue section for pathological score.

### BUN and Cr measurement

Blood clotted for 30 min at room temperature to precipitate the serum. Then isolated the serum (7500 rpm for 10 min at 4 °C) to measure the level of BUN and Cr. The level of BUN and Cr in serum were measured by 7180 Biochemical Analyzer (Hitachi, Japan).

### Western blotting

The lysates of kidney tissue and renal tubular epithelial cells were used to detect the proteins concentration. The standard procedure of Western blot was used as described, following with the used of these antibodies: Cx32 (1:1000; Abclonal); NLRP3(1:1000; Abcam); GSDMD (1:1000; CST); IL-18(1:1000; Abcam); β-actin (1:50000; Abclonal); Rabbit secondary antibody (1:20000; Abcam). All the Western blots were repeated for more than three times and the images were captured by Tanon 5500 imaging system (Tanon, Shanghai). All images were analyzed by the ImageJ scanning software and the level of proteins were expressed as the relative values to the sham or control groups.

### Immunofluorescence

The kidney sections of 5-μm thick embedded in paraffin and the treated TECs were used to detect the expression level of NLRP3 protein. Immunofluorescence followed the standard procedures as described with the use of the NLRP3 antibody in a ratio of 1:100 and 6-diamino-2-phenylindole (DAPI) was used to stain cell nuclei. All images were captured by the EVOS FL fluorescence microscope (EVOS FL, Life Technology).

### Cell culture and treatment

HK-2 cells and NRK-52E cells were obtained from American Tissue Culture Collection. HK-2 were cultured in Keratinocyte-SFM medium contained with 3% fetal bovine serum (FBS). NRK-52E cells were cultured in the Dulbecco’s Modified Eagle’s medium (DMEM) supplement with 10% FBS. Cells were grown at 37 °C in the relatively sterile incubator (Eppendorf, Hamburg, Germany) with 5% CO2 in air.

HK-2 cells and NRK-52E cells H/R models were used to mimic the renal I/R injury as previously described [23]. Briefly, the treated cells were cultured in the hypoxia chamber (Galaxy 48 R; Eppendorf, Hamburg, Germany), containing 95% N2 and 5% CO2, for 24 h before re-oxygenating by exposing to incubator, where containing 95% air and 5% CO2, for 4 h (H24R4). Cells in the control groups didn’t suffer from as previously described [22], cells were seeded at two kinds of density to decide whether to form the GJ channels, one is at low density (25,000 cells/cm2, no GJ formed), the other is at high density (125,000 cells/cm2, GJ formed). 70 to 80% of confluent cells was the prerequisites to go on the next step. At high density HK-2 cells were separated into the following groups: H/R group, group of MR (MCC950 (10 μM, 1 h) before H/R), group of 2APB (pretreated with 2APB of 25 μM before H/R).

### Cell viability and cytotoxicity assay

Cells were planted in 96-well plate to detect the viability and cytotoxicity of each group by using a CCK-8 assay kit, according to the manufacturer’s instructions.

### Scanning electron microscopy

The treated cells were fixed with 2.5% glutaraldehyde for 1 h at the room temperature, and at the 4 °C temperature environment for 3 h. After that, pyrophosphate buffer was used for the secondary fixation. Subsequently, cells were dehydrated using gradient ethanol and acetate. The samples were observed under scanning electron microscopy (hitachi S-3000 N, Japan) after staining with gold.

### Cell transfection

Cx32-siRNA (:5′-CCGGCATTCTACTGCCATT-3′;

5′-GAAGAGGUAUUGAAUGCUA-3′) duplexes targeting Cx32 human gene (NCBI Gene ID: 2705) were used to silence the expression of Cx32. A nonspecific siRNA was used as control group (NC group). As for the overexpression and knockdown of miR-155-3p, the mimics、inhibitor and their negative controls were obtained from RiboBio. Transfection into HK-2 cells were carried out by using transfection kit (Ribobio, China), according to the manufacturer’s protocols. For Cx32-OP, HK-2 cells were plated at high density (125,000 cells/cm^2^) in 6-well plates. A total of 4 μg Plasmid-Cx32 was mixed with Lipo8 000™ transfection reagent to add to the medium. After 48 h, expression of Cx32 was detected by western blot analysis. HK-2 cells were transfected with 4 μg empty vector served as the control group.

### ELISA

Levels of IL-18 and IL-1β in renal tissue and cells culture supernatant were detected by using the ELISA kits (Abclonal, China). The procedures were carried out by following the manufacturer’s instructions.

### RNA isolation and RT-qPCR

Total RNA was extracted from kidney tissues by Trizol reagent (Thermo Fisher Scientific, Invitrogen, USA). Subsequently, a total of 10 ng RNA was used for cDNA synthesis and the RT-qPCR reactions were performed by C11030–1 riboSCRIPT mRNA/lncRNA RT-qPCR Starter Kit (RiboBio, China). Bulge-Loop™ miRNA RT primer Set and mRNA RT primer Set (RiboBio, China) were used for miR155-3p and NLRP3 respectively. U6 and GAPDH were used as the internal standard for gene expression respectively. The relative quantitative was calculated by using the 2^−ΔΔCT^ method.

### FISH

Cells seeded on the coverslip were fixed by 4% paraformaldehyde and then digested by proteinase K. Next, cells were incubated with pre-hybrid solution at 37 °C for 1 h after. After that, probes hybridized at 4 °C overnight and then washed by sodium citrate buffer before staining nuclei with DAPI. The images were observed and collected under fluorescence microscope after dropping anti-fluorescence quenching agent.

### Statistical analysis

All data were analyzed using Prism Software (GraphPad 8.0). Normally distributed data were expressed as mean ± S.D., Student’s t test and one-way analysis of variance was used to compare the differences among groups. Differences were considered to be statistically significant at *P* < 0.05.

## Discussion

**Fig. 7:**
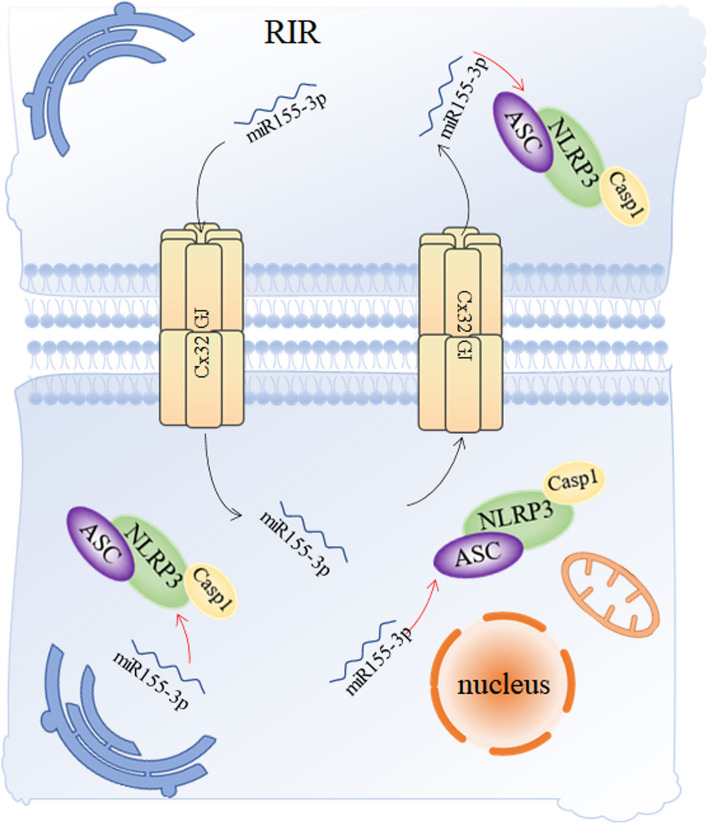
Correlation between Cx32 and its GJ and TECs' pyroptosis signaling pathway during renal I/R. In renal IR-induced AKI, damaged renal tubular cells transmit miR155-3p to neighboring cells through Cx32-GJ, which leads to NLRP3-mediated renal tubular epithelial pyroptosis and aggravates renal injury. When the function of Cx32- GJ is inhibited, the transmission of miR155-3p between cells is weakened, pyroptosis is inhibited, and renal injury is effectively alleviated. RIR: renal ischemia reperfusion; ASC: apoptosis-associated speck-like protein containing a caspase recruitment domain; Casp1: caspase 1

Renal I/R caused a high incidence and mortality of AKI, and the underlying mechanism is still not clarified. According to current data, it showed that tubules pyroptosis acted as a promoter in renal I/R injury and inhibition of NLRP3-mediated pyroptosis alleviated severity of AKI after I/R; Cx32 played a regulatory role in AKI and NLRP3-related pyroptosis, as element protein of GJ among renal cells, and meanwhile it enhanced the signaling of miR155-3p after I/R or H/R. Besides, decline of miR155-3p improved both NLRP3-realted tubular pyroptosis and AKI. In conclusion, these results indicated that signal pathway of Cx32/miR155/NLRP3 served as a central regulator of tubular pyroptosis and AKI after renal I/R, which was the first time to report the correlation between TECs’ intracellular GJ and tubules pyroptosis during renal I/R, and it was modified by the bystander effect of GJ about delivery of miR155-3p among adjacent cells. This study put forward a new direction of AKI treatment and preventation (Fig. 7).

As mentioned above, cell pyroptosis contributes to AKI induced by I/R. Canonical pyroptosis was mediated by inflammasomes consist of a sensor, an adaptor and an effector component such as caspase 1, and possibly caspase 11 (mouse) and caspase 4 and 5, among which NLRP3 inflammasome complex is the most common inflammasome. NLRP3-inflammsome activation leads to cell swelling by GSDMD, and release of mature pro-inflammatory cytokines, typically IL-1β and IL-18 [40, 41]. Our results represented significant depression of GSDMD, GSDMD-N, IL-1β and IL-18 when MCC950 was used to inhibit the level of NLRP3 inflammasome in kidneys of I/R-induced AKI, which confirmed that NLRP3-inflammasome mediated pyroptosis caused by renal I/R injury, as supported by other studies [14, 17, 19]. Nevertheless, Shigeoka et al. found that NLRP3 instead of NLRP3 -inflammasome took part in TECs pyroptosis and I/R-injury [42], and this different pattern of NLRP3 in AKI might be due to the variety of experimental models. Bilateral renal pedicles of mice were clipped for 45 min of ischemia while it was only 25 min in studies of Shigeoka. To deeply analyze the difference of data, on one hand, renal veins contained some oxygen for cell respiration, and on the other hand, shorter ischemia time led to milder kidney injury, which might not be capable for activation of the formation of inflammasome for pyroptosis. Beyond that, another research found during kidney injury NLRP3 was activated in an inflammasome- independent manner without maturation of caspase-1 and secretion of IL-1β and IL-18, and it mainly mediated the regulation of mitochondria [43]. This suggested diverse role of NLRP3 in kidney injury after I/R and the detailed mechanism needed further exploration.

According to our previous study, Cx32 - GJ aggravated renal tubular epithelial cell apoptosis and expanded AKI by mediating excessive ROS transmission during renal ischemia reperfusion [22, 44]. Similarly it was found in this experiment that inhibition of Cx32-GJ both prevented kidney damage after I/R or H/R and decreased the level of NLRP3,GSDMD,IL-1β and IL-18. Besides, by transfected with Cx32-plasmid in HK-2 cells, Cx32 overexpression enhanced expression of NLRP3,IL-1β and IL-18 while depressed cells survival after H/R. Furthermore, NRK-52E cells line in low and high density was used to detect the levels of GSDMD, NLRP3 and IL-18 after H/R, and the results showed the similar results in HK-2 cells that inhibition of Cx32-GJ (both at low cell density and 2-APB pretreatment) made the NRK-52Es suffer less H/R damage and cellular pyroptosis. These data indicated that Cx32 and its GJ had a regulatory effect on NLRP3-pyroptosis pathway during I/R-AKI, apart from ROS mediated-apoptosis signaling in tubular cells, which was never reported before in I/R-AKI. Other studies have also confirmed that GJ played a part in cell pyroptosis. After liver I/R injury, Cx32 expression was upregulated. Cx32 regulated oxidative stress and induced inflammatory response by interacting with PKC-α to regulate NF-κB/NLRP3 [45]. In addition, downregulation of Cx43 restrained NLRP3-inflammasome activation in LPS-induced kidney injury [46]. The role of GJ on cell death was modulated by multiple factors including the nature of connexin isoforms, cell types, and types of small molecules that were allowed to pass through GJ and so on [24]. Therefore, it raised our interest to explore the regulator role of Cx32-GJ in tubule pyroptosis under condition of I/R, and we proposed it was modified by specific molecules transferred through GJ channels.

Going through literature it was substantiated small molecular substances of less than 1 kDa, including microRNA, can freely diffuse between cells through GJ [47]. Janani Saikumar et. studied the miRNA profile that increased in the kidney after acute renal I/R injury and confirmed miR155 was one of the miRNAs that specifically increased and closely related to inflammation and respondence to cytokine stimulation after I/R injury [48]. As is known, miR-155 and its mature fragments miR155-3p and miR155-5p are perfectly suitable for passing through channels composed of Cx32 protein. In the HK-2 H/R model, we found that miR155-3p expression was significantly reduced compared to the other two miRNAs after Cx32 inhibition. Similarly, in kidney slices of wild mice after renal IR miR155-3p expression significantly increased while depressed in mice of Cx32^−/−^ or inhibiting GJ function with 2-APB. Therefore Cx32-GJ mediated transmission of miR155-3p content during renal I/R injury. In addition, after using miR155-3p mimics, HK-2 cells survival was downregulated while cell pyroptosis increased, confirming that miR155-3p participates in pyroptosis and AKI caused by I/R. Studies have confirmed that in early inflammation stage, miR155-3p responded quickly to the stimulations based on its positive feedback mechanism related with NF-kB/AP − 1 transcription factor [37, 49]. Another research also verified that in murine microglial cells miR155-3p was related with NLRP3 inflammasome activation and inflammation, which was consistent to our notion [35]. Nevertheless, Wang et al. [50] confirmed that miR-92a-3p was another important regulator of renal tubular epithelial cells pyroptosis and inhibiting miR-92a-3p expression reduced NLRP3, caspase-1, GSDMD-N, IL-1β and IL-18 expression. Whether other microRNAs except miR155-3p involved in regulation of pyroptosis is worth discussing. In conclusion, it was deserved to mention that pathway of Cx32-GJ-miR155-3p in tubule pyroptosis added to our knowledge of the role of Cx32 in AKI, and provided a new signaling pathway on cell death during AKI.

However, the study still had limitations. Firstly further in vivo experiments that overexpressing Cx32 in the renal tubular cells are needed to make the results more persuasive. Secondly, more tubular epithelial cell lines to investigate the role of miR155-3p on tubular pyroptosis in vitro would help to confirm the current results. Moreover, in order to investigate the role of miR155-3p in pyroptosis and AKI, additional in vivo experiments by knockdown or overexpression of miR155-3p are necessary in the future.

In summary, this study revealed that Cx32-related GJ bystander effect promoted development of renal inflammation and AKI induced by renal I/R via transmitting miR155-3p. This experiment further confirmed the role of pyroptosis in AKI, and proposed that Cx32-miR155-3p had a significant effect in early stage of AKI and tubules pyroptosis, which provided new ideas for early treatment and prevention of AKI after I/R.

### Supplementary Information

**Additional file 1.**

**Additional file 2.**

## Data Availability

The original contributions presented in the study are included in the article/ Supplementary Material further inquiries can be directed to the corresponding authors.

## Abbreviations

GJ: gap junction
Cx32: connexin 32
I/R: ischemia reperfusion
AKI: acute kidney injury
HK-2: human kidney tubular epithelial cells
NRK-52E: rat kidney tubular epithelial cells
ELISA: enzyme-linked immuno sorbent assay
FISH: fluorescence in situ hybridization
GSDMD: Gasdermin D
TLRs: toll-like receptors
NLRP3: NLR family pyrin domain containing 3
IL-1β: Interleukin-1β
ROS: reactive oxygen species
TECs: tubule epithelial cells
miRNA: microRNA
Cr: creatinine
BUN: blood urea nitrogen
H/R: hypoxia reoxygenation
Cx32^−/−^: Cx32 knockout
WT: wild type
RT-qPCR: Quantitative real-time PCR
2APB: 2-Aminoethoxydiphenyl borate
H&E: hematoxylin–eosin staining
DMEM: Dulbecco’s Modified Eagle’s medium
Cx32-OP: Cx32 overexpression
DAPI: 4′,6-Diamidino-2-Phenylindole
