# Supplementary material for: Connexin32 gap junction channels deliver miR155-3p to mediate pyroptosis in renal ischemia-reperfusion injury
Source: Cell Commun Signal. 2024 Feb 12;22:121. doi: 10.1186/s12964-023-01443-3 (PMC10863161; doi:10.1186/s12964-023-01443-3)
Supplement: Supplementary file 2 — Additional file 1. [file 12964_2023_1443_MOESM1_ESM.docx]

**Groupings**

**Experiments *in vivo***: Wild type of mice were divided into 8 groups (n=5 per group): sham group, group of 1h(sacrificed at 1h after reperfusion), group of 6h, group of 12h, group of 24h, group of 48h, group of 72h, group of MR(pretreated with 50mg/kg MCC950 for 1h before IR and sacrificed at 24h after reperfusion),group of 2APB(pretreated with 2APB 20mg/kg i.p. for 1h before renal ischemia and sacrificed at 24h after I/R). Cx32 gene knockout (Cx32^-/-^) mice were divided into 5 groups (n=5 per group): sham group, group of 1h, group of 6h, group of 12h and group of 24h.

**Experiments *in vitro***: HK-2 cells were totally divided into 14 groups: control group of LD (cells were cultured at low cell density), control group of HD(cells were cultured at high cell density), HR group of LD, HR group of HD, group of 2APB(pretreated with 2APB of 25μM before HR at high cell density), group of Cx32 siRNA(Cx32-siRNA of 50nM transfected into cells for 48h before HR at high cell density), group of Cx32-OP(cells were transfected with plasmid-Cx32 for 48 hours before HR at high cell density),group of mimic(miR155-3p mimic (50nM) transfected into cells for 48 hours before HR at high density),group of Inhibitor(miR155-3p inhibitor (100nM) transfected into cells for 48 hours before HR at high density), group of MR(MCC950 (10μM, 1h) before HR),control group of 2APB( pretreated with 2APB of 25μM before next step at high cell density), control group of Cx32-siRNA(Cx32-siRNA of 50nM transfected into cells for 48h before next step at high cell density), control group of Cx32-OP(cells were transfected with plasmid-Cx32 for 48 hours before next step at high cell density),control group of mimic(miR155-3p mimic (50nM) transfected into cells for 48 hours before next step at high density), control group of inhibitor(miR155-3p inhibitor (100nM) transfected into cells for 48 hours before next step at high density), n=5.
