# Supplementary material for: Connexin32 gap junction channels deliver miR155-3p to mediate pyroptosis in renal ischemia-reperfusion injury
Source: Cell Commun Signal. 2024 Feb 12;22:121. doi: 10.1186/s12964-023-01443-3 (PMC10863161; doi:10.1186/s12964-023-01443-3)
Supplement: Supplementary file 3 — Additional file 2. [file 12964_2023_1443_MOESM2_ESM.docx]

**Supplementary Figure S1. MCC950 alleviates IL-18 and mature IL-18 expression in renal tissue after I/R and in HK-2 cells undergoing H/R.**

The kidneys in mice subjected to renal I/R were harvested 24 hours after renal reperfusion. HK-2 cells were seeded at high density (125,000 cells/cm2, GJ formed). Cells in the H/R group were exposed to hypoxia for 24 hours and reoxygenation for 4 hours.

（A）The expression of IL-18 and mature IL-18 was analyzed by western blot using kidney lysates. * p<0.05 vs. sham; # p<0.05 vs. I/R. Data are means ± SEM (n=3).

（B）The expression of IL-18 and mature IL-18 in HK-2 cells was analyzed by western blot. * p<0.05 vs. control; # p<0.05 vs. H/R. Data are means ± SEM (n=3).





**Supplementary Figure S2. Survival Rate of HK-2 Cells in Different Density Groups.**

（A）HK-2 cells were seeded at low density(25,000 cells/cm2, no GJ formed) and high density (125,000 cells/cm2, GJ formed). Cells in the hypoxia/reoxygenation (H/R) group were subjected to 24 hours of hypoxia followed by 4 hours of reoxygenation. LD: low density group; HD: high density group. *p<0.05 vs. the control group at high density; # p<0.05 vs. the H/R group at low density; & p<0.05 vs. the control group at low density. Data are presented as the mean ± SEM (n =5).





**Supplementary Figure S3. Effects of reagents on the survival rate, IL-18，and IL-1β levels of HK-2 cells.**

(A) The survival rate of HK-2 cells in control groups with different regimens.

(B) Concentration of IL-18 and IL-1β in the culture supernatant of HK-2 cells in control groups with different regimens. Con: control group; 2APB: control cells pretreated with 2APB (25μM) for 1 hour; siRNA: Cx32-siRNA (50nM) transfected into control cells for 48 hours; Cx32-OP: cells transfected with plasmid-Cx32 for 48 hours to achieve overexpression of Cx32 before the next step; Mimic: mimic of miR155-3p (50nM) transfected into control cells for 48 hours; Inhibitor: inhibitor of miR155-3p (100nM) transfected into control cells for 48 hours. Data are presented as means ± SEM (n=5).





**Supplementary Figure S4. Inhibition Cx32 channel function attenuates NLRP3-mediated pyroptosis in H/R treated HK-2 cells.**

HK-2 cells were seeded at high density (125,000 cells/cm2, GJ formed). Cells in the hypoxia/reoxygenation (H/R) group were subjected to 24 hours of hypoxia followed by 4 hours of reoxygenation.(A) Representative images for the Western blot analysis of NLRP3, GSDMD, GSDMD-N, IL-18, and mature IL-18 in HK-2 cells. (B-F) Quantitative analysis of NLRP3, GSDMD, GSDMD-N, IL-18, and mature IL-18 in Western blots. Con: control group; 2APB: cells pretreated with 2APB (25μM) for 1 hour before the next step; siRNA: Cx32-siRNA (50nM) transfected into cells for 48 hours before the next step. * p<0.05 vs. control; # p<0.05 vs. H/R. Data are presented as means ± SEM (n=3).





**Supplementary Figure S5. Inhibition Cx32 channel function attenuates NLRP3-mediated pyroptosis in H/R treated NRK52E cells.**

NRK-52E cells were seeded at low density (25,000 cells/cm2, no GJ formed) and high density (125,000 cells/cm2, GJ formed). Cells in the hypoxia/reoxygenation (H/R) group were subjected to 24 hours of hypoxia followed by 4 hours of reoxygenation.

（A)The expression of NLRP3, GSDMD, GSDMD-N, IL-18 and mature IL-18 in NRK-52E cells were analyzed by western blots. LD: low density group; HD: high density group. *p<0.05 vs. the control group at high density; # p<0.05 vs. the H/R group at low density; & p<0.05 vs. the control group at low density. Data are means ± SEM (n =3).

(B) Survival rate of NRK-52E cells at different density. LD: low density group; HD: high density group. *p<0.05 vs. the control group at high density; # p<0.05 vs. the H/R group at low density; & p<0.05 vs. the control group at low density. Data are presented as the mean ± SEM (n =5).

(C) Survival rate of NRK-52E cells at high density in different groups. * p<0.05 vs. control; # p<0.05 vs. H/R. Data are means ± SEM (n = 5).

(D) Representative images and the relative expression of NLRP3, GSDMD, GSDMD-N, IL-18, and mature IL-18 in NRK52E cells analyzed by western blots. Con: the control group; 2APB: cells were pretreated with 2APB (25μM) for 1h before next step. * p<0.05 vs. control; # p<0.05 vs. H/R. Data are means ± SEM (n =3).





**Supplementary Figure S6. miR155-3p promotes NLRP3 expression in H/R treated HK-2 cells.**

(A) Immunofluorescence results of NLRP3 expression in HK-2 cells. Con: control group; Mimic: miR155-3p mimic (50nM) transfected into cells for 48 hours before the next step; Inhibitor: miR155-3p inhibitor (100nM) transfected into cells for 48 hours before the next step. Bar = 50μM.
